# What should the doctor prescribe—formula diet or antidiabetics? Effectiveness of formula diet-based lifestyle intervention vs. pharmacological antiglycemic therapy on weight loss and HbA1c reduction in type 2 diabetes patients—a systematic review

**DOI:** 10.3389/fendo.2025.1644442

**Published:** 2025-10-02

**Authors:** Kerstin Kempf, Martin Röhling, Stephan Martin

**Affiliations:** ^1^ West-German Centre of Diabetes and Health, Düsseldorf Catholic Hospital Group, Düsseldorf, Germany; ^2^ Faculty of Medicine, Heinrich-Heine-University Düsseldorf, Düsseldorf, Germany

**Keywords:** systematic review, type 2 diabetes, GLP-1 RA, SGLT-2 inhibitor, weight loss, formula diet, meal replacement

## Abstract

**Aim:**

Lifestyle intervention is the basis in type 2 diabetes therapy and leads, combined with formula diet, to substantial improvements in body weight and glycemic control up to diabetes remission. However, pharmacological therapies have also shown promising results. The aim of this systematic review was to compare the effects of large-sized formula diet-based lifestyle interventions vs. pharmacological interventions with glucagon-like peptide 1 receptor agonists (GLP-1 RAs), GLP-1, and glucose-dependent insulinotropic polypeptide (GIP) combinations and sodium glucose cotransporter-2 (SGLT-2) inhibitors on weight and HbA1c reduction in obese type 2 diabetes patients.

**Methods:**

Literature searches were performed using PubMed for articles published until February 5, 2025. Primary and secondary outcomes were changes in weight [kg] and HbA1c [%] determined as estimated treatment difference (ETD) of intention-to-treat analyses (with a treatment policy approach).

**Results:**

Of 1,409 identified articles, 54 articles describing 3 formula diet-based lifestyle interventions as well as 47 randomized, placebo-controlled pharmacological studies met our inclusion criteria including n=87.871 patients (32.8 ± 1.7 kg/m², 60 ± 4 years, 43 ± 7% women). Formula diet-based lifestyle intervention might more strongly reduce weight compared with pharmacological interventions with GIP/GLP-1 RAs or SGLT-2 inhibitors after <12 months (studies’ mean values: −5.6 vs. −2.6 kg) or ≥12-month intervention periods (−7.3 vs. −3.1 kg). Despite a trend for treatment superiority of pharmacological therapies in the short term (−0.9 vs. −0.6%), long-term HbA1c reduction was comparable between lifestyle and pharmacological interventions (−0.7%).

**Conclusions:**

There is evidence that formula diet-based lifestyle intervention might improve weight loss to a greater extent than pharmacotherapies with comparable long-term glycemic control. Thus, formula diet-based lifestyle intervention might be a valid therapy option for obese patients with type 2 diabetes.

## 1 Introduction

Weight reduction in overweight type 2 diabetes patients contributes to improvements in glycemic control, reduces the risk for cardiovascular and renal events, and has beneficial effects on mortality and diabetes-related comorbidities (1). Weight loss can be stimulated by different non-pharmacological (2–4) or pharmacological approaches (5). Lifestyle intervention, as part of the first-line therapy for type 2 diabetes, is one of the cornerstones in the management but also prevention of type 2 diabetes comprising diet, physical activity, and further healthy behaviors (6). A high certainty of evidence had been found for the beneficial effects of formula diet-based lifestyle intervention on improving body weight (7) and glycemic control (2) and has recently been incorporated into the current ADA guidelines as a valid option for the treatment of type 2 diabetes (1). However, lifestyle alterations fall entirely within the responsibility of those affected and are rarely supported by the health system in terms of personnel or finances. In contrast, when type 2 diabetes cannot be managed with a behavioral approach to achieve glycemic targets (HbA1c <7.0%), pharmacological intervention is needed (8), which in turn is paid for by the health system. In this context, selective glucagon-like-peptide-1 receptor agonists (GLP-1 RAs) (5) or dual glucose-dependent insulinotropic polypeptide (GIP) and GLP-1 RA therapy (9) as well as sodium-glucose cotransporter 2 (SGLT2) inhibitors have shown promising results regarding weight loss and glycemic control.

While formula-based lifestyle interventions were originally developed to reduce weight, the primary aim of pharmacological interventions is to improve glucose control. Nevertheless, randomized-controlled trials have shown that both types of intervention support weight loss and HbA1c reduction. However, a comparison of effects has not systematically been reviewed so far.

It is therefore the purpose of this review to summarize systematically the effects of large study-sized non-pharmacological and pharmacological interventions on weight loss and glycemic control in obese patients with type 2 diabetes focusing on the therapy approaches of formula diet-based lifestyle intervention, selective GLP-1 RAs, and dual GIP/GLP-1 RAs as well as SGLT-2 inhibitors.

## 2 Methods

### 2.1 Study design

This review was based on PRISMA (Preferred Reporting Items for Systematic Reviews and Meta-analysis) guidelines (10) (**Supplementary Table 1**). In this systematic review, the effect of formula-diet based lifestyle interventions has been examined. Formula diets were defined as meal replacements substituting main meals with prepackaged, nutritionally complete products like powders, shakes, or soups. They are composed of simple substances that do not require digestion, are readily absorbed, and leave a minimum residue in the intestine. Formula diets are generally designed to provide a balanced intake of nutrients, focusing on calorie restriction for weight loss. Furthermore, based on the current ADA guidelines (8) grading the “weight change” potential of current antiglycemic drugs with “loss”, the most weight loss-potential drugs (the first three GLP-1 RAs (semaglutide, liraglutide, and exenatide) and the first three SGLT-2 inhibitors (empagliflozin, dapagliflozin, and canagliflozin)), based on a comprehensive meta-analysis (5), which was also cited in the ADA guideline statement in this regard, were included into the present review. Additionally, the most promising new substance class, the dual GIP/GLP-1 RA (tirzepatide), was also included into the analysis.

### 2.2 Search strategy and data sources

Literature searches were performed using PubMed until February 5, 2025. Search terms used were as follows: (type 2 diabetes) AND (exenatide OR liraglutide OR semaglutide OR tirzepatide OR formula diet OR meal replacement OR “low-calorie diet” OR canagliflozin OR dapagliflozin OR empagliflozin) AND (HbA1c OR glycosylated hemoglobin A OR A1c OR blood glucose OR “weight loss” OR weight) AND (placebo (for drug-related studies)) in article title and abstract. Reference lists of reviews as well as meta-analyses and all included articles identified by the search were also examined for other potentially eligible studies.

### 2.3 Eligibility criteria (participants; interventions; comparators)

Studies that met the following criteria were included in this review: (i) published in English; (ii) sample size of intervention (verum) and control groups should be ≥100 persons per group at baseline to reduce the possibility for a publication bias; (iii) study population should be obese (BMI: ≥30 kg/m²; as the primary outcome is absolute weight change), diagnosed with type 2 diabetes, and adult (≥18 years); (iv) for pharmacological studies: a randomized and placebo-controlled study design that did not include an active add-on cotreatment like an additional new drug was mandatory; (v) for non-pharmacological studies: studies with formula diet-based lifestyle intervention should be compared with standard care (with conventional diet or a less intense approach); and (vi) measurements of changes in body weight and HbA1c should be available. Pharmacological and non-pharmacological interventions that did not last for at least 20 weeks to investigate the chronic and long-term effects on body weight and glycemic control were excluded. *Post-hoc* analyses, pooled analyses, and phase 1 and 2 studies were also not considered.

### 2.4 Study sections and data extraction

After removal of duplicates, study and data extraction were performed independently by two of the investigators based on the predetermined criteria using the software EndNote X8, and conflicting data were decided by a third independent investigator. The first investigator listed the reported ETDs in body weight and in HbA1c in a table; values have been controlled by the second investigator. If studies published more than one article with different time points, all relevant data were included.

### 2.5 Data analysis

Due to the methodological differences, especially in terms of study design and the comparability problem between non-pharmacological and pharmacological studies, we decided to not conduct a meta-analysis and thus only summarize the means and error-related variations (e.g., standard deviation (SD) and confidence interval (CI)) of the intervention effects from each included study. Clinically relevant improvements of body weight or glycemic control were defined as ETDs of ≥5% in weight (1) or 0.6% in HbA1c (8). Study effects reported at different time points within each study were stratified into <12 months or ≥12 months to differentiate between mid-term and long-term effects. To prevent overestimation of effects, only longitudinal data were considered when an intention-to-treat (ITT) approach was applied to determine effect sizes. Thus, in the case of pharmacological studies, the considered analysis method was the treatment policy approach (e.g., used in the PIONEER 1 study (11)) as it broadly corresponds with the aforementioned ITT analysis approach. In the case of different dosages examined in one study, only the outcome of the larger dose was compared with the placebo outcome. Summary measures were differences in absolute changes following the intervention in body weight (in kg, primary outcome) and HbA1c (in %, secondary outcome). Studies reporting sufficient data to calculate estimated treatment difference (ETD) were considered for the review. Where not reported, changes and treatment differences were calculated (12).

### 2.6 Quality and bias assessment

To evaluate potential study bias, study quality was assessed according to the Grading of Recommendations, Assessment, Development and Evaluations (GRADE) system (73). Risk of publication bias in this investigation is estimated graphically by funnel plots. A reverse funnel shape of the effect size distribution of the included interventions represents an unbiased distribution.

### 2.7 Systematic review protocol

Not publicly available.

## 3 Results

### 3.1 Study selection and population characteristics

**Figure 1:**
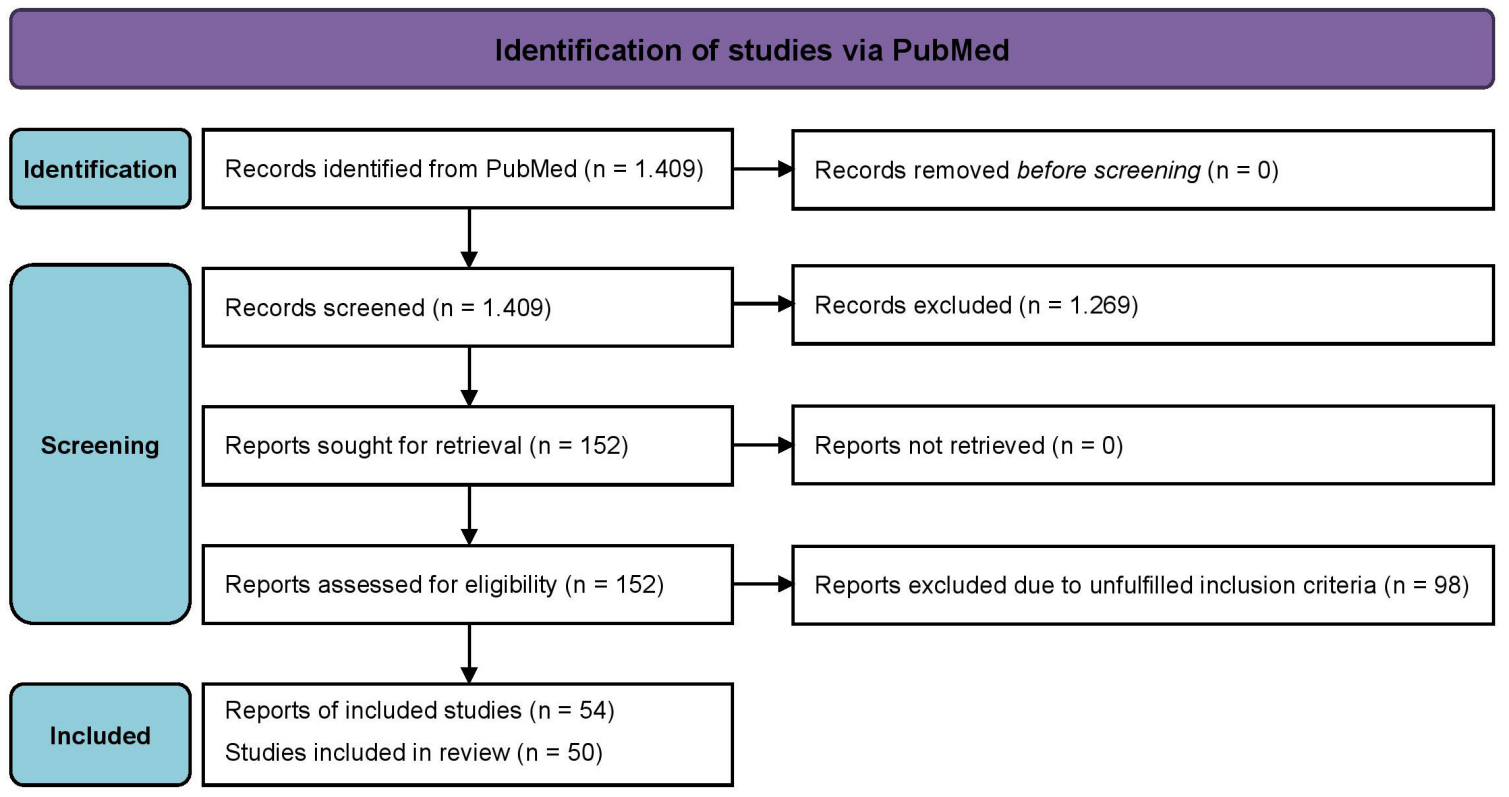
PRISMA 2020 flow diagram.

Of 1,409 identified articles, 54 articles reporting 50 trials, i.e., 3 formula diet-based lifestyle interventions as well as 47 randomized and placebo-controlled pharmacological studies, met our inclusion criteria (**Figure 1**). A total of n=87.871 patients were included into the final analysis (with mean study values for BMI: 32.8 ± 1.7 kg/m², age: 60 ± 4 years, sex: 43 ± 7% women; HbA1c: 8.2 ± 0.3%). For the observation period of <12 months or ≥12 months, n=35 or n=24 trials with n=27.066 or n=79.025 participants were analyzed (n=9 studies reported outcomes at both observation periods). **Table 1** provides further insights into each included study in this review stratified by type of intervention and/or type of drug class. All three treatment options (formula diet-based lifestyle intervention: 10%; GIP/GLP-1 RAs or GLP-1 RAs: 17%; SGLT-2 inhibitors: 16%) had comparable studies’ mean dropout rates in the verum group.

**Table 1:** Baseline characteristics of the included studies.

Trial	Intervention	Intervention group [n]	Control group [n]	Sex (female) [%]	Diabetes duration [years]	Age [years]	Weight [kg]	BMI [kg/m²]	Hba1c [%]	Data <12 months	Data ≥12 months
Lifestyle interventions											
TeLiPro (13)	Formula diet	102	100	46	11	60	107	36.0	8.3	x	x
DiRECT (15)		150	149	40	3	54	100	34.6	7.6		x
LOOK AHEAD (14, 29)		2,570	2,575	59	4	59	101	36.0	7.3		x
Studies’ mean values		943	941	48	6	58	103	35.5	7.7		
GIP/GLP-1 RAs											
SURPASS-1 (30)	Tirzepatide	121	115	59	5	53	85	31.6	8.0	x	
SURPASS-5 (16)		120	120	45	13	60	95	33.3	8.3	x	
SUSTAIN 1 (31)	Semaglutide	130	129	43	4	54	93	33.0	8.1	x	
SUSTAIN 5 (32)		131	133	44	14	59	91	32.0	8.3	x	
SUSTAIN 6 (33)		1,648	1,649	39	14	65	92	32.8	8.7		x
SUSTAIN 9 (34)		151	151	42	10	57	92	31.9	8.0	x	
PIONEER 1 (11)		175	178	49	4	54	88	32.0	8.0	x	
PIONEER 4 (19)		285	142	48	8	57	93	32.7	8.0	x	x
PIONEER 5 (35)		163	161	52	14	70	91	32.4	8.0	x	
PIONEER 6 (36)		1,591	1,592	32	15	66	91	32.3	8.2		x
PIONEER 8 (37)		181	184	46	15	61	86	31.0	8.2	x	x
STEP 2 (18)		404	403	49	8	55	100	35.5	8.1		x
STEP-HFpEF DM (17)		310	306	43	8	70	103	36.9	6.8		x
LIRA-RENAL (38)	Liraglutide	140	137	49	15	67	95	34.0	8.1	x	
LIRA-ADD2SGLT2i (39)		203	100	40	10	55	91	32.2	8.0	x	
LEAD-1 (40)		234	114	49	7	55	83	30.1	8.5	x	
LEAD-2 (41)		242	122	40	8	57	na	31.2	8.4	x	
LEAD-4 (42)		178	177	43	9	55	na	33.7	8.5	x	
LEAD-5 (43)		230	114	45	9	58	86	30.8	8.3	x	
SCALE Insulin (44)		198	198	48	12	57	100	35.5	8.0		x
SCALE Diabetes (45)		423	212	50	7	55	106	37.2	7.9		x
LEADER (46)		4,668	4,672	36	13	64	na	32.5	8.7		x
NN2211-3917 (47)		226	225	42	12	58	91	32.3	8.2	x	
EXENATIDE-112 (48)	Exenatide	113	113	46	6	53	100	34.0	8.2	x	
EXENATIDE-113 (49)		129	123	40	6	56	96	34.0	8.7	x	
EXENATIDE-115 (50)		241	247	42	9	56	na	34.0	8.5	x	
NCT00765817 (51)		137	122	44	12	59	94	33.4	8.4	x	
DURATION-7 (52)		232	231	52	11	58	94	33.6	8.5	x	
EXSCEL (53)		7,356	7,396	38	12	62	92	31.8	8.0	x	x
Studies’ mean values		702	675	45	10	59	93	33.0	8.2		
SGLT-2 inhibitors											
EMPA-REG Basal (54)	Empagliflozin	155	170	44	na (>5 y)	59	93	32.3	8.3		x
EMPA-REG Renal (55)		187	187	43	na (>10 y)	65	83	30.3	8.1	x	x
EMPA-REG MDI (56)		189	188	57	na (>10 y)	57	96	34.8	8.3		x
EMPA-REG Outcome (57)		2,342	2,333	28	na (>10 y)	63	87	30.6	8.1		x
Study 05 (58)	Dapagliflozin	109	109	50	9	61	89	32.0	8.2	x	
Study 006 (61, 62)		196	197	47	14	59	94	33.1	8.5	x	x
NCT00528879 (21, 59)		135	137	44	6	54	87	31.3	8.0	x	x
NCT01031680 (63)		455	459	32	12	63	93	32.8	8.1	x	
NCT01042977 (64)		480	482	33	13	64	94	32.8	8.1	x	x
DELIGHT (60)		145	148	29	18	65	na	30.2	8.5	x	
DERIVE (70)		160	161	43	14	66	90	32.0	8.2	x	
DECLARE-TIMI 58 (65)		8,582	8,578	37	11	64	na	32.0	8.3		x
CANA (M+S)* (22)	Canagliflozin	107	106	43	10	57	92	32.0	8.5	x	
NCT01106690 (20)		115	114	33	11	58	94	32.7	8.0	x	
NCT01106651 (66, 67)		236	237	43	11	63	90	31.7	7.8	x	x
NCT01106677 (71)		367	183	53	7	55	86	31.4	8.0	x	
CREDENCE (68)		2,202	2,199	34	16	63	na	31.4	8.3		x
CANVAS (69)		5,795	4,347	36	14	63	90	32.0	8.2		x
Studies’ mean values		1,220	1,130	41	12	61	91	32.0	8.2		

BMI, body mass index; CON, control (placebo) group; INT, intervention (verum) group; na, not available; y, years; *self-chosen.

### 3.2 Synthesized findings

#### 3.2.1 Formula diet-based lifestyle interventions

**Figure 2:**
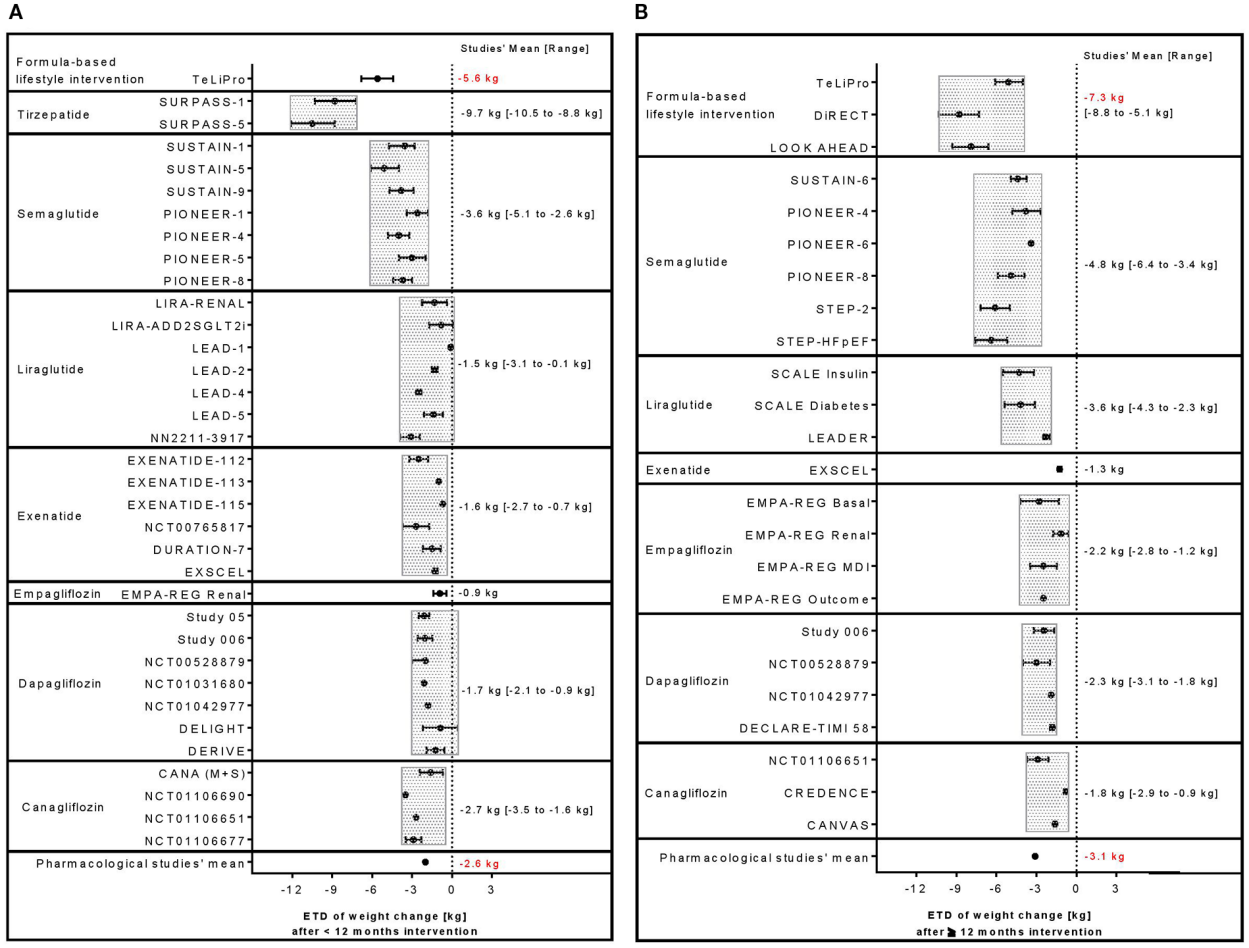
Comparison of ETD in weight change between formula diet-based lifestyle interventions with pharmacological therapies including selective GLP-1 or dual GIP/GLP-1 RA agent as well as SGLT-2 inhibitor studies reporting outcomes **(A)** <12 or **(B)** ≥12 months.

**Figure 3:**
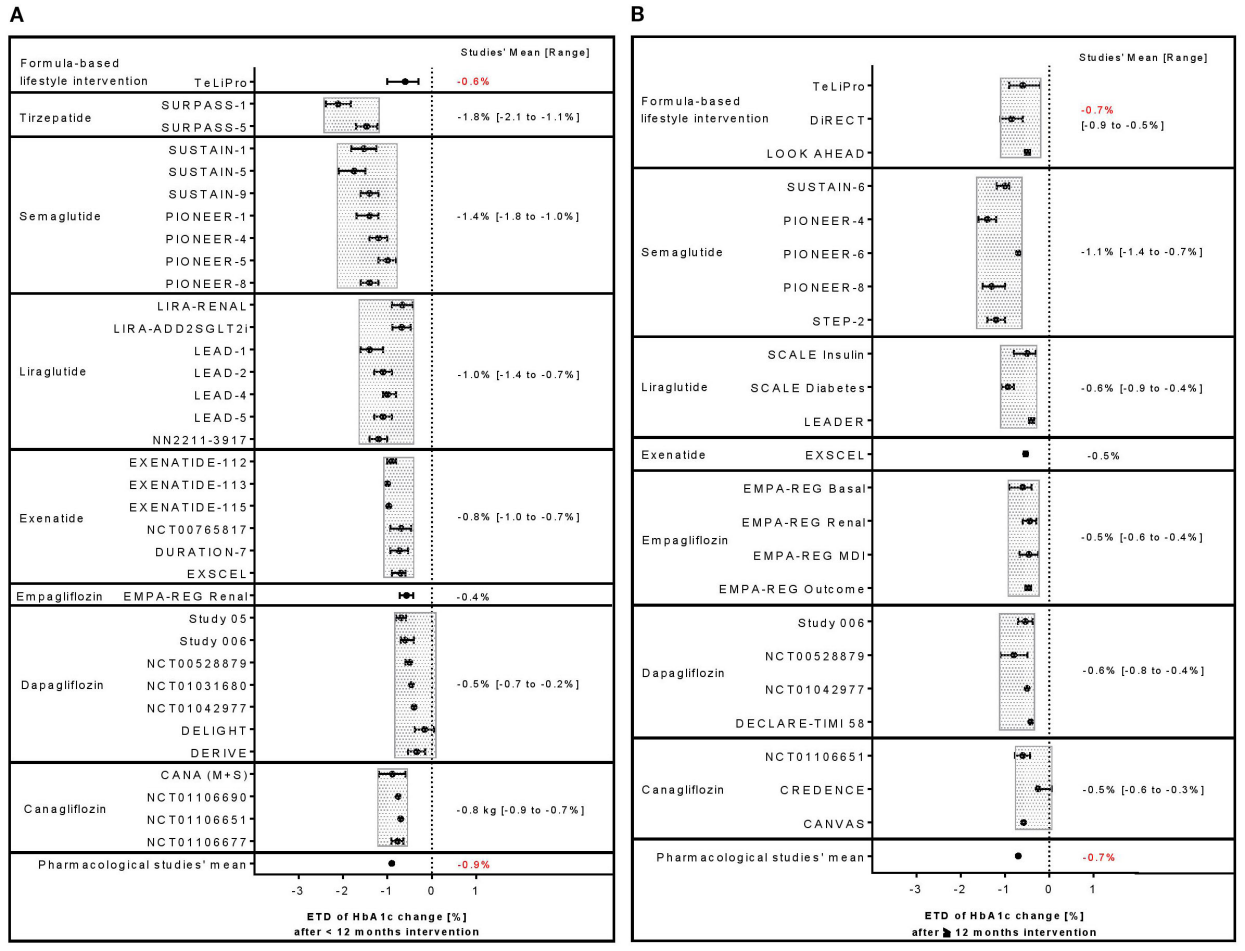
Comparison of ETD in HbA1c change between formula diet-based lifestyle interventions with pharmacological therapies including selective GLP-1 or dual GIP/GLP-1 RA agent as well as SGLT-2 inhibitor studies reporting outcomes **(A)** <12 or **(B)** ≥12 months.

Using formula diets as an integral part of a lifestyle intervention can result in meaningful improvements of body weight and glycemic control. Body weight reductions could be shown in one study with an observation period <12 months resulting in an ETD of −5.6 kg (13) (**Figure 2A**) or between −5.1 and −8.8 kg in studies (13–15) with a follow-up period ≥12 months (**Figure 2B**). Largest reductions were seen in TeLIPro (ETD −5.6 kg, <12 months (13)) and in DiRECT (−8.8 kg, ≥12 months (15)). HbA1c was also clinically relevant reduced by TeLIPro (13) with an ETD of −0.60% (<12 months; **Figure 3A**) and ETDs ranging from −0.50% to −0.85% in the long term (≥12 months), respectively (**Figure 3B**). Largest HbA1c ETDs were seen in TeLIPro (−0.60%, <12 months (13)) and DiRECT (−0.85%, ≥12 months (15)).

#### 3.2.2 Pharmacological studies with selective GLP-1 RA and dual GIP/GLP-1 RAs

Application of selective GLP-1 RAs or dual GIP/GLP-1 RA agents can result in clinically relevant improvements of body weight and glycemic control. A large range of body weight reductions could be observed ranging between ETDs of −0.10 and −10.5 kg or −1.30 and −6.4 kg in studies with a treatment period <12 months (**Figure 2A**) or ≥12 months (**Figure 2B**). Largest ETDs were seen in tirzepatide (−10.5 kg, <12 months (16)) and semaglutide (−6.4 kg, ≥12 months (17). Also, HbA1c was reduced with ETDs from −0.7% to −2.1% (<12 months; **Figure 3A**) and from −0.40% to −1.40% (≥12 months), respectively (**Figure 3B**) (18). Largest ETDs were seen in tirzepatide (−2.1%, <12 months (30)) and semaglutide (−1.4%, ≥12 months (19)).

#### 3.2.3 Pharmacological studies with SGLT2 inhibitors

Treatment with SGTL-2 inhibitors led to small or moderate, but constant reductions in body weight with ETDs ranging between −0.9 and −3.5 kg or −0.9 and −3.1 kg in studies with a treatment period <12 months (**Figure 2A**) or ≥12 months (**Figure 2B**). Largest ETDs were seen in canagliflozin (−3.5 kg, <12 months (20)) and dapagliflozin (−3.1 kg, ≥12 months (21)). Glycemic control could be improved in several studies with ETDs ranging from −0.2% to −0.9% (<12 months; **Figure 3A**) and from −0.3% to −0.8% (≥12 months), respectively (**Figure 3B**). Largest HbA1c improvements were shown in canagliflozin (ETD −0.9%, <12 months (22)) and dapagliflozin (−0.8%, ≥12 months (21)).

#### 3.2.4 Clinically relevant improvements of body weight or glycemic control

Clinically relevant improvements were defined as ETDs of ≥5% in weight. 100% of studies reporting formula diet-based lifestyle interventions fulfilled this criterion as well as 16% (data <12 months) and 40% (data ≥12 months) of the studies with selective GLP-1 RAs or dual GIP/GLP-1 RA agents but none of the studies using SGTL-2 inhibitors (**Table 2**). A clinically relevant ETD of 0.6% in HbA1c had been reached in the short term by 100% of lifestyle intervention and GIP/GLP-1 RA studies, 50% of trials with SGTL-2 inhibitors, 67%, 70%, and 36% in the long term.

**Table 2:** Number of studies reporting a clinically relevant improvements of body weight or glycemic control.

Intervention	Studies reporting an ETD in weight ≥5% [n]				Studies reporting an ETD in HbA1c ≥0.6% [n]			
	Data <12 months		Data ≥12 months		Data <12 months		Data ≥12 months	
Formula diet	1 of 1	100%	3 of 3	100%	1 of 1	100%	2 of 3	67%
Tirzepatide	2 of 2	16%	na	40%	2 of 2	100%	na	70%
Semaglutide	1 of 7		4 of 6		7 of 7		6 of 6	
Liraglutide	0 of 5		0 of 3		7 of 7		1 of 3	
Exenatide	0 of 5		0 of 1		6 of 6		0 of 1	
Empagliflozin	0 of 1	0%	0 of 4	0%	0 of 1	50%	1 of 4	36%
Dapagliflozin	0 of 6		0 of 3		2 of 7		1 of 4	
Canagliflozin	0 of 4		0 of 2		4 of 4		2 of 3	

Clinically relevant improvements were defined as ETDs of ≥5% in weight or 0.6% in HbA1c.

### 3.3 Assessment of quality and risk of bias

**Figure 4:**
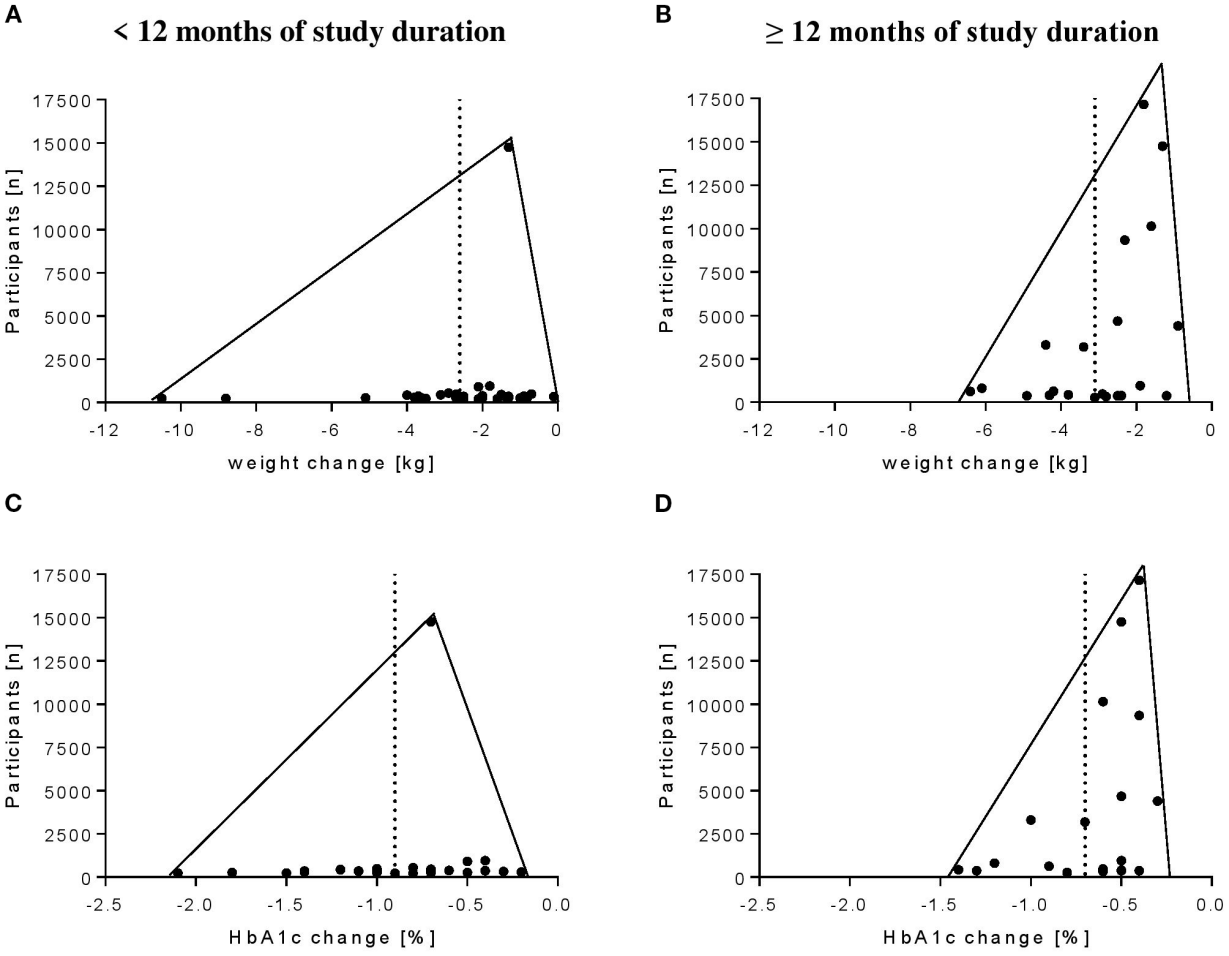
Funnel plots of pharmacological studies with selective GLP-1 or dual GIP/GLP-1 RA agents as well as SGLT-2 inhibitors. Shown are the ETDs of **(A)** weight change after <12 months of study duration, **(B)** weight change after ≥12 months of study duration, **(C)** HbA1c change after <12 months of study duration, and **(D)** HbA1c change after ≥12 months of study duration.

Results of assessment of study bias according to GRADE (73) are shown in **Supplementary Table 1**. Our assessment revealed no consistent patterns of bias across the included studies, and regardless of intervention type, overall certainty of evidence was moderate to high. Funnel plots were exploratorily created for pharmacological studies. All of them showed a left shift (**Figure 4**) for weight loss and HbA1c reduction (determined as ETD) after <12 and ≥12 months.

## 4 Conclusions

The present review indicates that formula diet-based lifestyle interventions are not inferior to pharmacotherapies in weight reduction and long-term glycemic control. Thus, formula diet-based lifestyle intervention might be considered as valid add-on therapy option for obese patients with type 2 diabetes instead of sole antidiabetic medication therapy.

Lifestyle intervention is the first-line therapy for type 2 diabetes. The potential of formula diet-based lifestyle intervention in improving body weight (7) and glycemic control has been recognized (2), resulting in the incorporation into the current ADA guidelines as a valid option for the treatment of type 2 diabetes (1). In the short as well as in the long-term formula diet-based lifestyle interventions demonstrated stronger weight reduction compared with pharmacological interventions with GIP/GLP-1 RAs or SGLT-2 inhibitors and comparable long-term HbA1c reduction. Lifestyle interventions are often criticized for their lack of sustainability compared with pharmacotherapy. However, this overlooks the fact that lifestyle interventions are only implemented for short periods, whereas drug treatment is lifelong and often with increasing dosages.

Tirzepatide showed the most promising weight loss in comparison with all other substance classes and treatment approaches in the observation period <12 months. Unfortunately, we did not identify long-term studies (≥12 months) of tirzepatide matching with our inclusion criteria. However, an active-comparator trial with tirzepatide vs. insulin glargine resulted in a clinically relevant weight loss with an ETD of −15.2 kg in obese patients with type 2 diabetes (BMI: 33.5 kg, n=358 vs. n=360) after 12 months (23), indicating long-term efficacy. The large weight loss in the present review was accompanied by clinically relevant HbA1c reductions as seen in other reviews (8). These improvements are likely to be promoted by direct and indirect actions on the pancreas (enhanced insulin secretion and reduced glucose-adjusted glucagon secretion). A further anorexigenic effect in the brain by activating signals of both GIP and GLP-1 receptor pathways accompanies this antiglycemic effect (24), resulting probably in this amount of weight loss.

Besides tirzepatide, the included GLP-1 RA trials resulted consistently in moderate to large effects regarding weight loss independently of the observation period. In detail, a more pronounced weight loss was shown for semaglutide in the long term (≥12 months), but liraglutide and exenatide had rather short-term efficacy (<12 months). These improvements were accompanied by clinically relevant changes in HbA1c with a pronounced effect by semaglutide regardless of the observation period. Mechanistic actions of GLP-1 RAs address probably similar pathways (25) comparable with the dual GIP/GLP-1 RA tirzepatide, however, with a smaller impact (24).

In contrast, all included SGLT-2 I trials showed only small to moderate effects on weight loss. This weight loss is primarily attributed to the study by Giugliano et al. (26). The difference in weight loss between GLP-1 RAs and SGLT-2 Is in the present work is in line with the findings of another review and meta-analysis (5). Tsapas et al. stated that semaglutide, exenatide, and liraglutide are more efficacious in reducing weight, followed by SGLT-2 Is. However, as shown in **Table 1** and in the results, the included population is trending to weighing less compared with the GLP-1 RA and GIP/GLP-1 RA participants.

Our review has several strengths and limitations. First, the number of included studies, especially for formula diet-based lifestyle interventions as well as pharmacological trials with tirzepatide (with a total of 476 patients), is low. However, this represents the situation in real life with favoring pharmacological interventions vs. lifestyle interventions. Since the lifestyle intervention studies included over 5,500 patients in total, this large number might strengthen the validity. Focusing on subgroups in regard to different dosages, application form (oral or subcutaneous) and observation period limit our ability to investigate into further insights. Second, in the formula diet-based lifestyle intervention studies, glucose-lowering medication was either adjusted in response to metabolic improvements due to the intervention (13, 14, 29) or even completely withdrawn before study start (15) so that the impact on HbA1c reduction as a secondary outcome may have been underestimated.

According to Grant and Booth (2009) (72), a meta-analysis requires that all studies should be similar, i.e., the population, intervention, and comparison, and that the same measure or outcome was measured in the same way at the same time intervals. Thus, the heterogeneity of the included non-pharmacological and pharmacological interventions with respect to design and population precluded a meaningful meta-analysis, so that just the calculation of studies’ mean values but not real summary estimates for treatment effects on weight loss and HbA1c has been possible. While this decision was made to maintain the validity of our findings, it is important to consider this aspect when interpreting the results.

The strengths of our study included the systematic approach in the retrieval of relevant large sample-sized studies and the clear focus on weight loss and HbA1c as outcomes because these represent the most common parameter to monitor treatment efficacy in obese patients with type 2 diabetes. The methodological quality of each study was independently rated by two investigators according to the GRADE criteria revealing no consistent patterns of bias across the included studies and overall moderate to high certainty of evidence, regardless of intervention type. Because of the strict inclusion criteria with regard to the characterization of the included studies, study population, and the main outcomes (weight loss and HbA1c), quality assessment was not used as an exclusion criterion. To reduce the probability of a publication bias, we only included studies into our review with a sample size of n ≥ 100 participants per group. Publication bias can be shown in funnel plots (**Figure 4**). In both, studies reporting results after <12 as well as ≥12 months there were a left shift toward higher reductions of weight and HbA1c in studies with smaller populations. Due to the small number of lifestyle studies, the funnel plot representation is not meaningful for them. Furthermore, to prevent further overestimation, we chose a rather conservative approach only considering outcome reportings with an ITT analysis. This type of analysis considers all patients that have started with the treatment or dropped out during the study. When comparing and interpreting the results, it should not be neglected that a few participant baseline characteristics differed between the treatment options.

On the one hand, one might argue that the weight loss potential of formula diet-based lifestyle interventions might be slightly overestimated as the baseline body weight and BMI trended to be larger compared with the pharmacological interventions (35.5 vs. 32.0 and 33.0 kg/m²). On the other hand, the antiglycemic effect of formula diet-based interventions then seems to be underestimated when considering the difference in baseline HbA1c (7.7% vs. 8.2%). Moreover, it should be taken into account for the overall study analysis that the included patient cohorts partly differed at baseline in terms of disease severity (diabetes duration and current diabetes therapy at baseline). However, the reported baseline values in **Table 1** represent the mean values of the intervention and control groups of the individual trials. Thus, in the formula-based lifestyle intervention trials, the baseline BMI has been higher in both the intervention and the control groups. Since in the present review only the ETDs were compared, different baseline values should not have any impact on effect size.

In sum, there are different potential antiglycemic treatment options targeting a reduction in body weight and glycemic control in obese patients with type 2 diabetes. Our review revealed that formula diet-based lifestyle interventions are not inferior to pharmacotherapies with GIP/GLP-1 RAs or SGLT-2 inhibitors. Moreover, with formula diet-based lifestyle interventions, a stronger weight reduction was seen after both <12 months and ≥12 months of intervention. Despite a trend for treatment superiority of pharmacological therapies in the short term, long-term HbA1c reduction was identical. Regarding dual GIP/GLP-1RAs, tirzepatide showed superior findings in comparison with all other agents or approaches, respectively. However, further research is needed to confirm the promising results in longer large study-sized trials (≥12 months).

Risk and grading of potential side effects, therapy compliance, and long-term efficacy of each treatment approach as well as patients’ preferences are crucial factors arguing for or against either strategy (8). However, these aspects are seldom referred to when comparing those approaches and should be considered when initiating a type 2 diabetes therapy. Despite the evident benefits of both treatment strategies and pharmacological or formula diet-based lifestyle interventions, for individuals with type 2 diabetes, further research is required to determine which subgroups of patients could benefit the most of either strategy. As part of the prevalent “precision medicine” initiative, future trials should address these groups to improve the understanding of either treatment option on weight loss and metabolic control. Furthermore, large sample-sized formula diet-based lifestyle intervention studies are missing, necessitating research in this field. In particular, existing large study-sized and long-term treatment investigations should be followed up to reveal substantial benefits from either therapy.

There is evidence that formula diet-based lifestyle intervention might improve weight loss to a greater extent than pharmacotherapies with comparable long-term glycemic control. Therefore, therapeutical decision making for the different treatment options should be weighed in broader context considering potential severe side effects of GLP-1 RAs (e.g., gastrointestinal events) or SGLT-2 Is (e.g., genital infections) (27), economic facets (28), and adherence to treatment regiments with formula diet-based lifestyle intervention as a valid (add-on) therapy option for obese patients with type 2 diabetes.

## Data Availability

The original contributions presented in the study are included in the article/**Supplementary Material**. Further inquiries can be directed to the corresponding author.
