# Supplementary material for: What should the doctor prescribe—formula diet or antidiabetics? Effectiveness of formula diet-based lifestyle intervention vs. pharmacological antiglycemic therapy on weight loss and HbA1c reduction in type 2 diabetes patients—a systematic review
Source: Front Endocrinol (Lausanne). 2025 Oct 2;16:1644442. doi: 10.3389/fendo.2025.1644442 (PMC12527879; doi:10.3389/fendo.2025.1644442)
Supplement: Supplementary file 1 [file Table1.pdf]

Supplementary Table 1. Quality assessment

Trial	Intervention	Risk of Bias	Inconsistency	Indirectness	Imprecision	Publication Bias	Overall Certainty
**Lifestyle interventions**							
TeLiPro [13]	Formula diet	Low to Moderate	Low	Low	Low	Low	High
DiRECT [15]		Low to Moderate	Low	Low	Low to Moderate	Low	Moderate to High
LOOK AHEAD [14, 29]		Low to Moderate	Low	Low	Low	Low	High
**GIP / GLP-1 RAs**							
SURPASS-1 [30]	Tirzepatide	Low to Moderate	Low	Low	Low to Moderate	Low	Moderate to High
SURPASS-5 [16]		Low to Moderate	Low	Low	Low to Moderate	Low	Moderate to High
SUSTAIN 1 [31]	Semaglutide	Low	Low	Low	Low	Low	High
SUSTAIN 5 [32]		Low to Moderate	Low	Low	Low to Moderate	Low	Moderate to High
SUSTAIN 6 [33]		Low	Low	Low	Low	Low	High
SUSTAIN 9 [34]		Low	Low	Low	Low	Low	High
PIONEER 1 [11]		Low to Moderate	Low	Low	Low to Moderate	Low	Moderate to High
PIONEER 4 [19]		Low	Low	Low	Low	Low	High
PIONEER 5 [35]		Low	Low	Low	Low	Low	High
PIONEER 6 [36]		Low	Low	Low	Low	Low	High
PIONEER 8 [37]		Low to Moderate	Low	Low	Low to Moderate	Low	Moderate to High
STEP 2 [18]		Low to Moderate	Low	Low	Low to Moderate	Low	Moderate to High
STEP-HFpEF DM [17]		Low	Low	Low	Low	Low	High
LIRA-RENAL [38]	Liraglutide	Low	Low	Low	Low	Low	High
LIRA-ADD2SGLT2i [39]		Low	Low	Low	Low	Low	High
LEAD-1 [40]		Low to Moderate	Low	Low	Low	Low	High
LEAD-2 [41]		Low to Moderate	Low	Low	Low to Moderate	Low	Moderate to High
LEAD-4 [42]		Low to Moderate	Low	Low	Low	Low	High
LEAD-5 [43]		Low to Moderate	Low	Low	Low to Moderate	Low	Moderate to High
SCALE Insulin [44]		Low to Moderate	Low	Low	Low	Low	High
SCALE Diabetes [45]		Low to Moderate	Low	Low	Low	Low	High
LEADER [46]		Low	Low	Low	Low	Low	High
NN2211-3917 [47]		Low to Moderate	Low	Low	Low to Moderate	Low	Moderate to High
EXENATIDE-112 [48]	Exenatide	Low to Moderate	Low	Low	Low	Low	High
EXENATIDE-113 [49]		Low to Moderate	Low	Low	Low to Moderate	Low	Moderate to High
EXENATIDE-115 [50]		Low to Moderate	Low	Low	Low	Low	High
NCT00765817 [51]		Low to Moderate	Low	Low	Low	Low	High
DURATION-7 [52]		Low to Moderate	Low	Low	Low	Low	High
EXSCEL [53]		Low to Moderate	Low	Low	Low to Moderate	Low	Moderate to High

Trial	Intervention	Risk of Bias	Inconsistency	Indirectness	Imprecision	Publication Bias	Overall Certainty
**SGLT-2 inhibitors**							
EMPA-REG Basal [54]	Empagliflozin	Low to Moderate	Low	Low	Low	Low	High
EMPA-REG Renal [55]		Low to Moderate	Low	Low	Low	Low	High
EMPA-REG MDI [56]		Low to Moderate	Low	Low	Low	Low	High
EMPA-REG Outcome [57]		Low	Low	Low	Low	Low	High
Study 05 [58]	Dapagliflozin	Low to Moderate	Low	Low	Low	Low	High
Study 006 [61, 62]		Low	Low	Low	Low to Moderate	Low	High
NCT00528879 [21, 59]		Moderate	Low	Low	Low	Low	Moderate to High
NCT01031680 [63]		Low	Low	Low	Low to Moderate	Low	Moderate to High
NCT01042977 [64]		Low	Low	Low	Low to Moderate	Low	Moderate to High
DELIGHT [60]		Low to Moderate	Low	Low	Moderate	Low	Moderate to High
DERIVE [70]		Low to Moderate	Low	Low	Low to Moderate	Low	Moderate to High
DECLARE-TIMI 58 [65]		Low	Low	Low	Low	Low	High
CANA (M+S)* [22]	Canagliflozin	Low to Moderate	Low	Low	Low to Moderate	Low	Moderate to High
NCT01106690 [20]		Low to Moderate	Low	Low	Low to Moderate	Low	Moderate to High
NCT01106651 [66, 67]		Low to Moderate	Low	Low	Low to Moderate	Low	Moderate to High
NCT01106677 [71]		Low to Moderate	Low	Low	Low to Moderate	Low	Moderate to High
CREDENCE [68]		Low	Low	Low	Low	Low	High
CANVAS [69]		Low	Low	Low	Low	Low	High

Criteria were rated according to the Grading of Recommendations, Assessment, Development and Evaluations (GRADE) system [73].
